# Ability to Care for an Ill Loved One During the First COVID-19 Lockdown: Mediators of Informal Caregivers’ Stress in Europe

**DOI:** 10.3389/fpsyt.2022.852712

**Published:** 2022-04-12

**Authors:** Shadya Monteiro, Margot Fournier, Jérôme Favrod, Anne-Laure Drainville, Léa Plessis, Sylvie Freudiger, Krzysztof Skuza, Charlene Tripalo, Nicolas Franck, Marie-Clotilde Lebas, Jocelyn Deloyer, Hélène Wilquin, Philippe Golay, Shyhrete Rexhaj

**Affiliations:** ^1^La Source, School of Nursing Sciences, HES-SO University of Applied Sciences and Arts Western Switzerland, Lausanne, Switzerland; ^2^Aix Marseille Université, LPCPP, Aix-en-Provence, France; ^3^AVASAD, Association Vaudoise d’Aide et de Soins à Domicile Route de Chavannes, Lausanne, Lausanne, Switzerland; ^4^HESAV, School of Health Sciences, HES-SO University of Applied Sciences Western Switzerland, Switzerland; ^5^Service de Psychiatrie Générale, Psychiatry Department, CHUV, Lausanne, Switzerland; ^6^Pôle Centre Rive Gauche et Centre Ressource de Réhabilitation Psychosociale et de Remédiation Cognitive, Centre Hospitalier Le Vinatier, UMR 5229, CNRS, Université Claude Bernard Lyon 1, Lyon, France; ^7^Département des Sciences de la Santé Publique et de la Motricité, Haute Ecole de la Province de Namur, Namur, Belgium; ^8^Centre Neuro Psychiatrique St. Martin, Dave Namur, Belgium; ^9^Community Psychiatry Service, Department of Psychiatry, Lausanne University Hospital and University of Lausanne, Lausanne, Switzerland

**Keywords:** informal caregiver, lockdown, COVID-19, perceived stress, mental health

## Abstract

Informal caregivers are overlooked, healthcare actors. They are at particular risk of distress and suffer from poor mental health. This study aimed to investigate the perceived stress and modulating factors during the first COVID-19 lockdown in Europe, regardless of the illness that care recipients suffer from. Sociodemographic data, coping resources, and perceived stress level using the Perceived Stress Scale (PSS-10) questionnaire were assessed using a web-based survey in Switzerland, France, and Belgium with 232 informal caregivers. Mediation analyses were used to identify the factors that modulate stress. Higher perceived stress among informal caregivers was associated with a younger age for the care recipient, family relationship with the care recipient, cohabitation, and female sex of the informal caregiver. These associations were partially mediated by the fear of getting ill (age, cohabitation), the conviction that lockdowns had a negative impact on health (age, kinship), and the perceived deterioration of the care recipient’s health (gender). The fear of losing the ability to cope with caregiving tasks due to an illness (COVID-19 and/or other) and the negative impact of the lockdown on care recipients’ health, particularly on the mental health of young care recipients, increased the stress of informal caregivers. Our results emphasize the importance of informal caregiving support to prevent heightened stress in lockdown conditions, regardless of care recipient illness or kinship.

## Introduction

In March 2020, the World Health Organization (WHO) declared the new coronavirus (COVID-19) outbreak to be a pandemic and called on countries to take action for population health and health services (1, 2). To reduce the risk of contagion, many European countries imposed containment measures during the coronavirus pandemic. Care consultations, day hospitals, and community facilities have quickly implemented strategies to protect patients from infection while providing routine care, such as teleconsultations (3, 4). However, restrictions on liberty, quarantine, and isolation have substantial, long-lasting, negative psychological impacts (5), potentially affecting more severely vulnerable populations. Informal caregivers help family members, friends, or neighbors to cope with disabilities or long-term illnesses and usually play an essential role in patient support. On the basis of studies in this domain, the term “informal caregiver” is applied to anyone who believes they have a caregiver role (6). Studies have shown that this role affects the quality of life, well-being, and mental health (7–11). The detrimental effect of poor informal caregiver health is twofold, as it also affects care recipients’ health (12, 13). During the first wave of the COVID-19 pandemic, informal caregivers were more often requested to (14–16) provide support without being able to rely on their specific usual support (17) due to a deterioration in the condition of their loved ones or because of the cancelation of certain healthcare services (18).

Studies have reported the general effects of pandemics on informal caregivers. Several studies have noted such psychological consequences as worry, stress, nervousness, and anxiety (19–23). The major concerns and stressors for informal caregivers are their own health and job loss. Being ill would impact greatly affects care and the possibility of transmitting COVID-19 (24). The fear of infecting their loved ones and the anticipated guilt should this happen have even led to greater avoidance of healthcare facilities among the informal caregiver population, be it for medical appointments or emergency care for themselves or their relatives (19). Moreover, informal caregivers were burdened by a new need to make decisions related to the health of their ill loved ones, on issues, such as symptom management or palliative care, to name but a few (21, 25). Some studies have also shown that informal caregivers can suffer as a result of their socioeconomic situations (18). The risk of contracting the virus at work or losing income could affect the level of care the ill person receives (18, 21, 26). The various consequences studied in the current scientific literature do not specify the perceived stress associated with how informal caregivers experience lockdown. However, it is important to clarify this link under high-pressure conditions to promote effective coping strategies for informal caregivers.

In this study, lockdowns are considered stress-triggering events as defined by Lazarus and Folkman (27): the person first interprets whether the stressor is a threat; should that be the case, the person evaluates the available strategies to cope with the event (27). The impact of a particular event on one’s health is not solely determined by the event’s inherent intensity; it depends on perception and personal and contextual factors (28). Perceived stress refers to an individual’s feelings or thoughts about the levels of stress that they experience at a given time or over a given period of time (29). The Perceived Stress Scale (PSS) developed by Cohen and Williamson evaluates whether the person feels able to cope with the event (28) and how the person perceives his or her control of the event (30). This study aimed to report the perceived stress of informal caregivers and the factors mediating this stress during the first COVID-19 lockdowns in Switzerland, France, and Belgium. We reported sociodemographic factors associated with perceived stress and identify mediators that modulate these associations, thus providing actionable points to alleviate stress among informal caregivers.

## Materials and Methods

### Design and Recruitment

This cohort study was an observational retrospective study targeting informal caregivers to assess their perceived stress, attitudes, and resources during the first COVID-19 lockdown. Convenience sampling was used to collect data, and a link to the online survey was sent by the researchers to informal caregivers in the French-speaking part of Switzerland, France, and Belgium through various communication channels (e.g., social networks, family support associations, and clinical networks). Questionnaires about the lockdown were completed retrospectively at the end of the first wave, between May and October 2020, and the survey was closed to focus on the early phase of the pandemic. To be included, participants had to be over 18 years old; live in Switzerland, France, or Belgium; and be an informal caregiver of at least one person. Self-identification as an informal caregiver was chosen to make it possible for all informal caregivers to participate without attempting to control their involvement in direct care or support to the care recipient in the particular context of the pandemic. To be considered informal caregivers for the purpose of data analysis, participants were to provide valid information about at least three of the following four items concerning the care recipient: gender, age, illness duration, and diagnosis. No inclusion or exclusion criteria were applied to care recipients.

### Instruments

#### Online Survey

The web-based survey is a self-report questionnaire collecting three types of data, i.e., (i) sociodemographic data; (ii) level of perceived stress during the COVID-19 lockdown; and (iii) attitudes and resources during the first containment, which corresponds to factors that may modulate perceived stress. The survey was adapted from a study assessing the impact of containment measures in the general French population (31, 32). It was adjusted by the researchers to match the socio-health specificities of the three targeted countries: Switzerland, France, and Belgium.

The REDCap web application was used to build the survey and collect data anonymously.

#### Sociodemographic Data

Three categories of sociodemographic data can be distinguished (Table 1), i.e., (i) specific information related to the informal caregiver, (ii) housing conditions, and (iii) specific information related to the care recipient as provided by the informal caregiver.

**TABLE 1:** Sociodemographic variables and their association with perceived stress.

Variable name		Categories	Mean ± sd [range] or n (%)	PSS-10 score	Test (d.f)	*p*-value
**Informal caregivers, *n* = 232**						
	**Age		54.0 ± 13.9 [19–87]		***r*** = −**0.178**	***p* = 0.007**
	**Sex	female	184 (79%)	18.8 ± 0.5	***t*(228)** = 2.995****	***p* = 0.003**
		male	46 (20%)	15.2 ± 1.1		
		other	2 (1%)			
	Marital status	married, partnership	138 (59%)	17.9 ± 0.6	*F*(2,231) = 1.011	*p* = 0.366
		single	49 (21%)	19.5 ± 1.2		
		separated, widowed	45 (19%)	17.7 ± 1.1		
	Country	Switzerland	129 (56%)	18.0 ± 0.7	*F*(2,231) = 0.192	*p* = 0.826
		France	73 (31%)	18.6 ± 0.9		
		Belgium	30 (13%)	17.8 ± 1.4		
	Education level	Compulsory school	9 (4%)	19.7 ± 7.0	σ = 0.005	*p* = 0.940
		Secondary level	50 (22%)	17.7 ± 7.0		
		Tertiary level	173 (75%)	18.2 ± 7.7		
	Employment status*	employed	122 (53%)	19.0 ± 0.7	***F*(2,222)** = 3.148****	***p* = 0.045**
		retired, inactive	88 (38%)	16.6 ± 0.8		
		other	22 (9%)	19.8 ± 1.6		
	Occupational rate		81 ± 23 [15–100%]		*r* = −0.041	*p* = 0.653
	**Link to the care recipient	parent	71 (31%)	19.8 ± 0.8	***F*(5,231) = 3.361**	***p* = 0.006**
		spouse	49 (21%)	17.6 ± 1.0		
		offspring	47 (20%)	18.6 ± 1.1		
		sibling	40 (17%)	18.2 ± 1.2		
		relative	13 (6%)	16.3 ± 2.4		
		other	12 (5%)	10.9 ± 1.8		
**Housing conditions**						
	N of cohabitants				*r* = 0.101	*p* = 0.126
	**Living with the care recipient	no	138 (59%)	16.7 ± 0.6	***t*(230)** = –3.831****	***p* < 0.001**
		yes	94 (41%)	20.4 ± 0.7		
**Care-recipient**						
	**Age		54.0 ± 25.1 [7–99]		***r* = –0.212**	***p* = 0.001**
	Sex	female	94 (41%)	17.7 ± 0.8	*t*(227) = –0.691	*p* = 0.490
		male	135 (58%)	18.4 ± 0.6		
		other	3 (1%)			
	Illness duration		11.0 ± 9.2 [1–49]		*r* = 0.042	*p* = 0.536
	Class of disorder	psychiatric	104 (45%)	18.6 ± 0.7	*F*(2,231) = 0.822	*p* = 0.512
		neurological	12 (5%)	19.0 ± 2.1		
		physiological	28 (12%)	18.7 ± 1.5		
		other cases	31 (13%)	15.9 ± 1.5		
		>1 condition	57 (25%)	18.3 ± 1.0		

Values of the corresponding statistical test (degree of freedom) are displayed with the p-values. Significant tests are indicated in bold. *p-value < 0.05; **p-value < 0.01.

#### Perceived Stress Scale

The PSS-10 comprises 10 items to evaluate the frequency of stress during a defined period using a scale ranging from “never” to “very often” (28). We used the validated French version of the PSS-10 (33, 34). Wording was adjusted to refer to COVID-19 lockdowns as stress events and to assess perceived stress during the previous month. The PSS-10 score was calculated as previously described (33). Participants with more than one missing item were removed from the PSS-10 questionnaire. Remaining missing data were imputed using the corresponding median value.

#### Attitudes and Resources of Informal Caregivers

The survey assessed the informal caregivers’ experience of the lockdowns, their attitudes toward the care recipient, their resources to cope with containment, including social support, their economic situation, their health, and their perceptions of information provided on the virus (refer to Table 2 and Supplementary Material). Responses were measured using a 4- or 5-point Likert scale. These factors potentially modulate perceived stress, and we tested whether they mediated the association between stress and sociodemographic variables.

**TABLE 2:** List of attitudes and resources of informal caregivers and their association with PSS-10 scores.

Support to care recipient (during containment)			
	* Frequency of face-to-face interaction	**σ = 0.163**	***p* = 0.014**
	Frequency of phone calls	σ = −0.060	*p* = 0.417
	Frequency of interaction by text	σ = 0.072	*p* = 0.342
	Disruption of therapeutic follow-up	*t*(227) = −0.932	*p* = 0.352
	**Perceived deterioration of care recipient’s health	***t*(199) = −**3.875****	***p* = 1.45 × 10^–4^**
**Resources to cope with containment**			
	Support available	*t*(190) = −0.109	*p* = 0.913
	Words of relatives and friends	*t*(230) = –0.632	*p* = 0.528
	*Belief in a positive outcome	***t*(230)** = 2.517****	***p* = 0.013**
	*Knowledge and scientific progress	***t*(151)** = 2.129****	***p* = 0.035**
	Experience, ability to face difficulties	*t*(220) = −0.783	*p* = 0.434
	Community actions and support	*t*(230) = 0.726	*p* = 0.469
	**Possible positive impact on the planet	***t*(230)** = 2.99****	***p* = 0.003**
	**Possible positive impact at the individual level	***t*(230)** = 4.293****	***p* = 2.6 × 10^–5^**
**Personal economic situation (during containment)**			
	**Negative impact of containment on budget	**σ = 0.201**	***p* = 0.002**
**Health**			
	**Fear of falling ill	***F*(3,228) = 10.520**	***p* = 2 × 10^–6^**
	*Concerns about the access to masks, gel, etc.	**σ = 0.179**	***p* = 0.006**
	**Negative impact of containment on health	**σ = 0.622**	***p* = 4,5 × 10** ^–^ **^26^**
	Clarity of COVID-19-related information	*r* = −0.032	*p* = 0.625

Values of the corresponding statistical test (degree of freedom) are displayed with the p-values. Significant tests are indicated in bold. *p-value < 0.05; **p-value < 0.005.

### Statistical Analyses

The database (*n* = 250 entries) was processed as follows: (i) removal of cases not providing information on care recipients (*n* = 10); (ii) removal of duplicates (*n* = 4); (iii) exclusion of persons living neither in Switzerland, France, nor Belgium (*n* = 3); and (iv) removal of participants with > 1 missing item in the PSS-10 questionnaire (*n* = 1). Remaining missing data were imputed using the corresponding median value. For categorical sociodemographic variables, categories corresponding to less than 4% of the cohort were dropped (i.e., “other” for sex) or pooled (i.e., marital status, educational level, employment status, link to care recipient, and class of disorder). For potential mediators, we selected the clinically most meaningful in relation to stress and the cohort (e.g., more than 20% of the subjects answered at least two different items).

In the first step, the associations between perceived stress (PSS-10 score) and sociodemographic variables, on the one hand (Table 1), and potential mediators (Table 2), on the other, were assessed. For continuous variables (e.g., age), we tested the association with the PSS-10 score using Pearson’s correlation; for ordinal variables (e.g., education level), we tested the association with PSS-10 score using Spearman’s correlation; for categorical variables, we compared PSS-10 scores between groups using either Student’s *t*-test in the case of two groups (e.g., sex) or one-way ANOVA in the case of three groups or more (e.g., marital status). The tests were performed using SPSS version 25.

In the second step, mediation analyses for PSS-10 scores were performed to explore whether the relationship between perceived stress and the previously identified significant sociodemographic variables was mediated by other variables. Models were estimated separately for each previously identified potential mediator; for each model, the dependent variable was the PSS-10 score, and the independent variable was one of the sociodemographic variables. For the nominal variables “link to the care recipient” and “fear of getting ill,” we dichotomized the variables according to the best multinomial model using the exact likelihood with a uniform prior on all parameters (35).

Complete mediation was present when the path between PSS-10 scores and sociodemographic variables (i.e., the direct effect) was no longer significant after introduction of the mediator and the indirect effect was significant; partial mediation was present when the direct and indirect effects were statistically significant. Confidence intervals were estimated using bias-corrected bootstrapping with 1,000 draws. Mediation analyses were performed using Jamovi version 1.6.15 (36, 37).

### Ethical Considerations

The research protocol was approved by the local ethics committee of Switzerland (Commission cantonale d’éthique de la recherche sur l’être humain [CER-VD]), a member of Swissethics.

## Results

### Demographics

Study participants were informal caregivers (*n* = 232) who were recruited in Switzerland (56%), France (31%), and Belgium (13%) (Table 1). They were mainly women (79%), with a mean age of 54.0 ± 13.9 years. Most participants were relatives of their care recipients (parents, 31%; spouses, 21%; offspring, 20%; siblings, 17%; or other family relatives, 6%) and were living with the care recipients (59%).

Care recipients were mainly men (58%) with a mean age of 54 ± 25.1 years and diverse illnesses (e.g., psychiatric disorders, 45%; physiological pathologies, such as cancer, 12%; more than one diagnosis, 25%). The mean illness duration was 11 years (range, 1–49 years).

### Association Between Perceived Stress and Sociodemographic Variables

For the 14 sociodemographic variables (Table 1), we assessed the association with the level of perceived stress using the PSS-10. Six variables were robustly associated with the PSS-10, such as age of informal caregiver, sex of informal caregiver, employment status of informal caregiver, relationship with the care recipient, living with the care recipient, and age of care recipient. Informal caregivers had higher PSS-10 scores when their care recipients were younger, when they were relatives of the care recipient, or when they lived with him or her. Retired or inactive participants had lower PSS-10 scores than other occupational groups.

We further analyzed only the five most robust sociodemographic variables (*p* < 0.01) and tested whether some of them were interdependent; mediating models were computed to assess whether some of the variables concerning the care recipient (i.e., link to care recipient, living with care recipient, and age of care recipient) fully mediated the association between PSS-10 levels and the sociodemographic variables directly related to the informal caregiver (i.e., age and sex). The nominal variable “relation to care recipient” needed to be dichotomized for subsequent analyses, and the two categories retained were “family member” and “other relative,” according to the best multinomial model. Six models were tested, indicating that the age of the care recipient fully mediated the association between PSS-10 and age of the informal caregiver (*p* = 0.027, Supplementary Table 1). Therefore, the ages of the informal caregivers were not analyzed further.

In summary, four sociodemographic variables were retained for subsequent analyses, such as sex of informal caregiver, relationship with the care recipient, living with the care recipient, and age of care recipient.

### Selection of Variables Potentially Mediating the Perceived Stress

Participants rated their attitudes and resources during the first lockdown of the pandemic toward caregiving, resources, economic status, and health (Table 2). Statistical analyses indicated that 10 of the 18 corresponding items were associated with PSS-10. We applied a stringent cutoff to further analyze the most robust ones (*p* < 0.005). Six variables, such as perception of care recipient’s health deterioration, possible positive impact of containment on the planet, possible positive impact of containment at the individual level, negative impact of containment on one’s budget, fear of falling ill, and negative impact of containment on one’s health, were considered.

PSS-10 scores among informal caregivers were higher when they feared getting ill with COVID-19 and/or another disease, when they reported that the lockdown would likely have a negative impact on their health or budget, and when they felt that the health of their care recipient was deteriorating. In contrast, PSS-10 scores were lower when informal caregivers considered that a lockdown would have a positive impact at the individual level or for the planet. The nominal variable “fear of falling ill” was dichotomized to allow subsequent mediation analyses: the two categories retained were “yes” (fear of COVID-19 and/or another disease) and “no,” according to the best multinomial model.

In summary, six variables, such as perception of care recipient’s health deterioration, possible positive impact on the planet, possible positive impact at the individual level, fear of falling ill (dichotomized), negative impact of containment on one’s health, and negative impact of containment on one’s budget, which were related to resources or attitude were retained for subsequent analyses.

### Identification of Mediators for the Association Between Perceived Stress and Sociodemographic Variables

**FIGURE 1:**
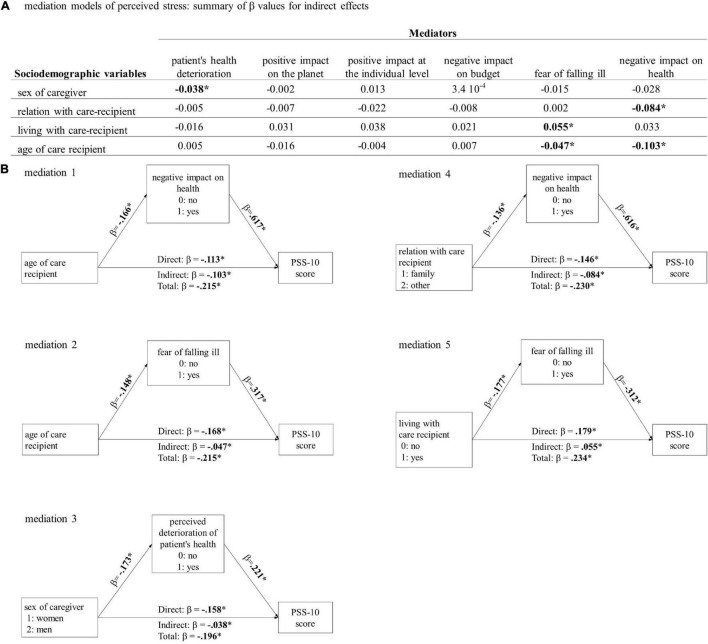
**(A)**. The standardized effect size (β) for indirect mediation of all the mediation models tested. Bold highlights *p*-value < 0.05. In all cases, direct effects are significant. **(B)**. Diagram illustrating the significant mediations and reporting the standardized effect size (β) of the corresponding effects. *p < 0.05.

For each of the four sociodemographic variables associated with PSS-10 scores, we tested six previously selected potential mediators (Figure 1A). As previously detailed, two nominal variables were dichotomized (i.e., link to care recipient and fear of falling ill); hence, mediations specific to some family members (e.g., parents vs. spouses) or to a particular illness were not assessed. Partial mediations were detected in five cases (Figure 1B). The relationship between younger age of the care recipient and higher PSS-10 score for the informal caregiver was partially mediated, on the one hand, by the conviction that lockdowns had a negative impact on health (Mediation 1), and on the other hand, by the fear of the informal caregiver of falling ill (Mediation 2). A higher PSS-10 mean among women than among men is partially mediated by an increase in the perceived deterioration of the care recipient’s health (Mediation 3). Increased perceived stress in informal caregivers who are family members of the care recipient vs. other relationships was partially mediated by the conviction that lockdowns have negative impacts on health (Mediation 4). Living together with the care recipient is associated with higher perceived stress of the informal caregiver, a relationship partially mediated by the fear of illness (Mediation 5).

## Discussion

This European study focused on informal caregivers to assess their perceived stress following the COVID-19 lockdown and identify the mediating factors. Perceived stress among informal caregivers was robustly associated with four sociodemographic factors (i.e., age of care recipient, family link to the care recipient, gender of the informal caregiver, and cohabitation), and these associations were partially mediated by three distinct attitudes of the informal caregivers. The relationship between increased perceived stress of the informal caregiver and lower age of the care recipient was partially mediated by informal caregivers’ fear of falling ill, on the one hand, and by the conviction that lockdowns have a negative impact on health, on the other. Compared with other relationships, increased perceived stress among informal caregivers who are family members of the care recipient was also partially mediated by the conviction that lockdowns have negative impacts on health. Higher perceived stress levels among women were partially mediated by an increase in the perceived deterioration of the care recipient’s health. The higher perceived stress level among caregivers living with the care recipient was partially mediated by fear of illness.

The first strength of this study is its international design, which covers Switzerland, France, and Belgium. Despite the diversity of the measures implemented and governmental communication strategies, we found no differences in perceived stress among informal caregivers in the three countries. A second strength is a focus on informal caregivers, regardless of the care recipient condition. Although the study participants faced a range of illnesses, we did not detect any effect of diagnosis on their perceived stress. Our results support the idea that guidance for informal caregivers should be provided independently of the patient’s diagnosis.

The sociodemographic factors associated with perceived stress among informal caregivers in this study are consistent with several reports: female sex, close kinship, cohabitation, and younger age are associated with burden, depressed mood, and health problems among informal caregivers (9, 38, 39). Furthermore, our data confirmed that these known risk factors were specifically modulated by lockdowns. Thus, the findings of a recent Italian study (40) can be extended to the three European countries included in this study. The analyses further clarified which attitudes and beliefs mediated these associations, thus providing actionable points to alleviate stress and burden among informal caregivers.

Interestingly, while Zucca et al. concluded that younger age of the informal caregiver of patients with dementia is a risk factor for higher stress in the context of lockdowns (40), our results show that this association is mediated by the younger age of the care recipient when various diseases are considered. Therefore, young age of the informal caregiver *per se* is not the only risk factor for increased stress. It might seem counterintuitive that the age of the care recipient is negatively associated with the level of perceived stress among informal caregivers, that is, that stress is higher when the care recipient is younger. In fact, it is not the at-risk population for COVID-19 complications (e.g., the elderly) that informal caregivers are most worried about, possibly because the health measures that are taken to control the pandemic also protected vulnerable persons. The mediation analyses highlighted two explanations for higher perceived stress results among informal caregivers with young care recipients: (i) informal caregivers’ fear of falling ill, possibly as she or he will be unable to cope with the caregiving tasks and (ii) the conviction that lockdowns have a negative impact on health, possibly as they perceived this problem early in the mental health of younger persons. However, several reports have warned about the negative impact of COVID-19 on the mental health of young people (i.e., children, adolescents, and students) in the early stages of the pandemic (41–43). Therefore, informal caregivers’ concern about the negative impact of lockdown on health for younger care recipients is likely to underlie mental health issues, but this point could not be disentangled based on the questionnaire. Interestingly, the conviction that lockdown had a negative impact on health also mediated the association of increased perceived stress in informal caregivers who were family members and those with other types of relationships. Family members might be more aware of the importance of mental resources for their care recipients than more distant relatives.

We reported that perceived stress in informal caregivers is higher for women than for men, which is consistent with reports covering the lockdown period (40, 44). In the general population, perceived stress due to the lockdown was also higher among women than among men, indicating that sex-specific factors were at stake (41, 42, 45). However, it is noteworthy that women were overrepresented in studies on informal care, arguably because they were more likely to self-identify as informal caregivers and engage in higher levels of care (9, 38, 46). In the context of the pandemic, the burden on informal caregivers was more likely to increase when it was initially high, and women were more likely than men to have an increased caregiving burden due to COVID-19 (47). This analysis further indicates that women’s perceived stress is partially mediated by a subjective perception that care recipients’ health is deteriorating, which may reflect their greater physical and emotional involvement in caregiving than what would be common among men.

Cohabitation is another major source of stress and burden for informal caregivers (40, 48–50). In the present cohort, 59% of the informal caregivers were living with their care recipients, and the fear of illness partially mediated their perceived stress. It is interesting to note that the fear of illness includes causes other than COVID-19 and, therefore, may involve the fear of not being able to manage their caregiving duties. Thus, the fear of contagion does not predominate when informal caregivers are considered globally (vs. when focusing on informal caregivers in contact with at-risk persons, such as the elderly) (40), whereas the fear of not coping with caregiving tasks is a shared concern. A Japanese study specifically indicated that 73% of caregivers of persons with schizophrenia worried about who would care for their patient if they became infected with COVID-19 (50). The link between cohabitation and stress, and the concern about not being able to meet the care recipient’s needs, may also be prevalent outside the pandemic context. This continuous strain places informal caregivers under pressure and is detrimental to their health.

The cross-sectional design of this study is a limitation that does not allow us to determine whether the impact of cohabitation was exacerbated by lockdowns. Another limitation linked to the study design is the retrospective collection of data; the level of perceived stress may change significantly as the situation evolves. The time window for the survey was limited to the end of the first wave to prevent bias as much as possible. The shortcoming of this pragmatic choice is the small cohort, which precluded more refined statistical analyses. Furthermore, the population targeted by the survey limits the generalizability of the results. Indeed, the channels used to share the survey (e.g., online social networks) specifically targeted informal caregivers in contact with informal caregivers’ associations or with health professionals. Study participants were better educated than is usually reported (51), suggesting that some groups were missed, possibly due to digital poverty or lack of health literacy skills. Moreover, the time spent providing care or support to the care recipient and the frequency of contact between the informal caregiver and the care recipient were not used as eligibility criteria because the pandemic context may have affected them drastically.

We showed that among informal caregivers, those who are relatives of the care recipient have reached similarly high levels of perceived stress regardless of the type of kinship (e.g., parents, siblings, or offspring). Moreover, we did not detect any effect of diagnosis on perceived stress, which is in agreement with other studies (40, 52). Overall, these findings underscore the importance of informal caregivers’ oriented support in the context of sanitary restrictions. With regard to Lazarus and Folkman, this study confirms the link between personal resources and the low level of stress perceived in this pandemic situation (27). For example, belief in a positive outcome is associated with lower levels of perceived stress, supporting the view that an optimistic attitude can improve the effectiveness of coping strategies (51). In practice, this result is favorable for tailored interventions for informal caregivers (53). Our findings suggest that supporting caregivers’ health and addressing the negative impact of the lockdown on mental health among young care recipients should be routinely included in intervention strategies to prevent heightened stress among informal caregivers.

## Data Availability Statement

The raw data supporting the conclusions of this article will be made available by the authors, without undue reservation.

## Ethics Statement

The studies involving human participants were reviewed and approved by the Commission Cantonale d’éthique de la Recherche sur l’être Humain (CER-VD). Written informed consent for participation was not required for this study in accordance with the national legislation and the institutional requirements.

## Author Contributions

SM, JF, and SR conceived the study. SM, JF, SR, and MF designed the study. SM, SR, JF, NF, HW, LP, M-CL, and JD developed the survey. SM, JF, LP, SF, KS, CT, NF, M-CL, and JD recruited the participants. MF, SR, and PG analyzed and interpreted the data. SM, MF, A-LD, and SR wrote the manuscript. All authors critically reviewed the final manuscript and approved the final version.

## Conflict of Interest

The authors declare that the research was conducted in the absence of any commercial or financial relationships that could be construed as a potential conflict of interest.

## Publisher’s Note

All claims expressed in this article are solely those of the authors and do not necessarily represent those of their affiliated organizations, or those of the publisher, the editors and the reviewers. Any product that may be evaluated in this article, or claim that may be made by its manufacturer, is not guaranteed or endorsed by the publisher.
