# Supplementary material for: Ability to Care for an Ill Loved One During the First COVID-19 Lockdown: Mediators of Informal Caregivers’ Stress in Europe
Source: Front Psychiatry. 2022 Apr 12;13:852712. doi: 10.3389/fpsyt.2022.852712 (PMC9039127; doi:10.3389/fpsyt.2022.852712)
Supplement: Supplementary file 2 [file Data_Sheet_1.PDF]

Supplementary Material. Questionnaire used in the survey to assess the attitudes and resources during the first containment of the participants. Italics correspond to the original French version. English is only presented here for convenience and was not used in the survey.

**Questions about support to care-recipient (during containment)**

During containment, how frequent were your interactions with your care recipient? (One response per option)
*Pendant cette période de confinement, à quelle fréquence avez-vous eu des interactions sociales avec votre proche malade ? (Une réponse par ligne)*

| | Never | Less than once a week | Once a week | Several times a week | Daily |
|---|---|---|---|---|---|
| | *Jamais* | *Moins d'une fois par semaine* | *Une fois par semaine* | *Plusieurs fois par semaine* | *Tous les jours* |
| - Face-to-face *Directement face à face* | | | | | |
| - Phone calls *Au téléphone* | | | | | |
| - Video-conference (Facetime, Skype, Whatsapp video,…) *En visio-conférence (Facetime, Skype, Whatsapp video,…)* | | | | | |
| - Text messages *Par messages (SMS, Whatsapp)* | | | | | |
| - On social media (Facebook, Instagram,…) *Sur les reseaux sociaux (Facebook, Instagram,…)* | | | | | |

| | | |
|---|---|---|
| Did the Covid-19 situation disrupt the usual therapeutic follow-up of your care recipient?<br>*La situation du Covid-19 a-t-elle perturbé le suivi thérapeutique habituel de votre proche malade ?* | Yes<br>*Oui* | No<br>*Non* |
| Do you perceive your care recipient's health as having deteriorated?<br>*Avez-vous l'impression que l'état de santé de votre proche s'est dégradé ?* | Yes<br>*Oui* | No<br>*Non* |

**Questions about resources to cope with containment**

| | | |
|---|---|---|
| Did you receive support during containment?<br>*Avez-vous eu du soutien durant cette période de confinement ?* | Yes<br>*Oui* | No<br>*Non* |

What helped you cope with confinement? (multiple answers)
*Qu'est-ce qui vous a aidé à supporter le confinement ? (plusieurs réponses possibles)*

| | Check |
|---|---|
| - Encouragement from your relatives and friends *Propos de votre entourage* | |
| - Commentary from the media *Propos des medias* | |
| - Belief in a positive outcome *Conviction d'une issue favorable* | |
| - Knowledge and scientific progress *Avancée des connaissances et progrès scientifiques* | |
| - Religious Faith *Foi religieuse* | |
| - Others non-Religious beliefs (magical thoughts; fortune telling; …) *Autres croyances non religieuses (pensées magiques; voyance;...)* | |
| - Experience and your ability to face difficulties Expériences passées et vos capacités à faire face aux difficultés | |
| - Accounts of similar experiences of others who were able to cope *Récits similaires personnes qui ont réussi à s'en sortir* | |
| - Community actions and support *Entraide et mise à disposition d'actions collectives* | |
| - Possible positive impact on the planet *Eventuelles répercussions bénéfiques que le confinement peut avoir sur la planète* | |
| - Possible positive impact at the individual level *Eventuelles répercussions positives du confinement à titre individuel* | |
| - Use of substances such as alcohol or cannabis *Recours à des substances comme l'alcool ou le cannabis* | |
| - Use of medication *Recours aux médicaments* | |
| - None of the above *Aucune de ces propositions* | |
| - Other *Autre* | |

| | | | | | |
|---|---|---|---|---|---|
| **Question about Personal economic situation (during containment)** | | | | | |
| Will containment have a negative impact on your budget?<br>*Cette période de confinement va-t-elle avoir des répercussions financières négatives sur votre budget (chômage, ...) ?* | Very likely<br>*Très probablement* | Likely<br>*Probablement* | Probably not<br>*Probablement pas* | Certainly not<br>*Certainement pas* | |
| **Question about health** | | | | | |
| Did you find the official coronavirus information clear enough (modes of transmission, prevention,…)?<br>*Avez-vous trouvé les informations officielles suffisamment claires sur le coronavirus (mode de transmission, prévention,…) ?* | Very clear<br>*Très claires* | Quite clear<br>*Assez claires* | Neither clear, nor<br>*Ni claires, ni floues* | Rather vague<br>*Plutôt floues* | Very vague<br>*Très floues* |
| Do you fear falling ill?<br>*Craignez-vous de tomber malade ?* | Yes, I fear falling ill with Covid-19<br>*Oui, je crains de tomber malade du Covid-19* | Yes, I fear falling ill with Covid-19 and with another disease<br>*Oui, je crains de tomber malade du Covid-19 et d'autre chose* | Yes, I fear falling ill with a disease other than Covid-19<br>*Oui, je crains de tomber malade d'autre chose que le Covid-19* | No, I do not have any particular fear<br>*Non, je n'ai pas de crainte particulière* | |
| Does the lack of access to personal protective equipment (masks, gel) concern you?<br>*Est-ce que le manque d'accès aux matériels de protection (masques, gel hydroalcoolique) vous préoccupe ?* | No<br>*Non* | Mostly no<br>*Plutôt non* | Mostly yes<br>*Plutôt oui* | Yes<br>*Oui* | |
| Will containment have a negative impact on your health?<br>*Cette période de confinement va-t-elle avoir des répercussions négatives sur votre santé ?* | Very likely<br>*Très probablement* | Likely<br>*Probablement* | Probably not<br>*Probablement pas* | Certainly not<br>*Certainement pas* | |
